# Porous ZnO Nanosphere Inherently Encapsulated in Carbon Framework as a High-Performance Anode For Ni–Zn Secondary Batteries

**DOI:** 10.3389/fchem.2022.936679

**Published:** 2022-06-30

**Authors:** Zhuo Li, Xianwei Hu, Jian Kang, Xiaoli Wang, Lingyu Kong, Zhongning Shi, Zhaowen Wang

**Affiliations:** ^1^ Key Laboratory for Ecological Metallurgy of Multimetallic Mineral (Ministry of Education), School of Metallurgy, Northeastern University, Shenyang, China; ^2^ State Key Laboratory of Rolling and Automation, Northeastern University, Shenyang, China

**Keywords:** intrinsic regulation, porous carbon shell, zinc oxide, anode, Ni-Zn batteries

## Abstract

Nickel–zinc (Ni-Zn) secondary battery that is environmentally friendly and inexpensive has been regarded as a promising rechargeable battery system. However, the generation of deformation and dendrites of the traditional zinc anode during the cycling can cause capacity degradation and impede its practical application. Herein, we design a hierarchical ZnO nanosphere coated with an inherently derived ZIF-8 porous carbon shell (ZnO@C_ZIF-8_) using a simple controllable method. The conductive carbon shell and porous ZnO core can provide more active sites, allow the fast transfer of electrons, and buffer the volume expansion of the electrode effectively. Benefiting from the synergistic effect amid the inherently ZIF-8–derived carbon shell and ZnO core, ZnO@C_ZIF-8_ nanospheres exhibit a satisfying capacity of 316 mAh g^−1^ at a current density of 1 A g^−1^ after 50 cycles and an outstanding rate capacity when acting as the anode for a Ni-Zn secondary battery with merchant agglomerative Ni(OH)_2_ as the cathode. These results imply that the ZnO@C_ZIF-8_ nanosphere is a hopeful anode for a high-energy Ni-Zn secondary battery.

## Introduction

Energy demand is increasing as societies continue to develop. Fossil fuels have caused severe pollution of the environment, so the development of environmentally friendly and renewable rechargeable battery systems is becoming increasingly important (Lund, 2007; Dunn et al., 2011; Wang et al., 2016). Rechargeable battery systems such as lithium-ion and nickel–hydrogen have received extensive attention because they are environment friendly and have considerable capacity (Yu et al., 2008; Lu et al., 2015; Xu et al., 2016). However, most existing rechargeable battery systems have limitations that hinder their further development. For example, the operating temperature range of the nickel–hydrogen battery is limited, and it often confronts a low operating voltage (Li et al., 2018). Lithium-ion batteries have high manufacturing costs, and the matched organic electrolyte has serious safety problems, such as toxicity and possibility of explosion (Stock et al., 2018; Yan et al., 2018). Compared with these battery systems, the nickel–zinc (Ni-Zn) secondary battery is a better alternative energy storage system with great prospects because of advantages such as cheap cost, safety, environmental friendliness, and outstanding specific energy density (Li and Dai, 2014; Yuan et al., 2014; Sun et al., 2016).

The anode is an important part of the nickel–zinc battery. However, the traditional zinc anode used in the Ni-Zn secondary battery suffers from deformation, dendrite, and corrosion during the charge and discharge processes. This results in capacity degradation, which severely limits the development of the Ni-Zn secondary battery (Lan et al., 2007; Wu et al., 2009; Nakata et al., 2015; Guo et al., 2017; Chen et al., 2021). Researchers have performed many studies to solve problems including surface modification (Park et al., 2018; He et al., 2021), structural optimization (Zeng et al., 2019a), and the use of active additives to improve the performance of zinc anode (Xie et al., 2015; Yi et al., 2021). Among them, carbon-shell–coated zinc oxide (ZnO) materials have shown great application potential. The carbon layer is coated on the surface of ZnO, which not only inhibits the dissolution of ZnO but also improves the conductivity of the base material and results in a symmetrical dispersion of electrons on the surface of the ZnO particles (Feng et al., 2015; Xia et al., 2019; Zhou et al., 2020). Long’s group prepared carbon-coated ZnO through the ball-milling pattern using glucose as the carbon source (Long et al., 2013). The material exhibited great cycling performance when used as an anode for Ni-Zn secondary battery. Other researchers prepared the ZnO/carbon nanotube composites by controlling the vertical growth of ZnO on carbon nanotubes (Cui et al., 2019). The unique heterostructure can efficiently improve the contact surface between the electrode and electrolyte to promote ion transport (Huang et al., 2014; Li et al., 2017a; Zeng et al., 2020). However, these strategies are only applied to modify the surface of ZnO by directly introducing the carbon source, which decreases the contact surface between the carbon material and ZnO and incompletely restrains the growth of dendrites. Therefore, it is necessary to realize a carbon-coating strategy that inherently evolves on the surface of ZnO to further enhance the electrochemical performance of zinc anode materials.

Zeolitic-imidazolate frameworks (ZIFs) are novel 3D framework materials that have received wide attention due to their well-designed morphology, ordered pore structure, and high stability (Lin et al., 2020; Huo et al., 2021; Xu et al., 2022). The pyrolysis product of ZIFs is a porous carbon material with a considerable specific area and conductivity under anaerobic conditions (Jiang et al., 2017; Li et al., 2020). Based on the aforementioned summary, we successfully synthesized a unique hierarchical ZnO nanosphere coated with ZIF-8 inherently derived porous carbon shell (ZnO@C_ZIF-8_) by using a simple hydrothermal method following pyrolysis. The electrochemical properties of ZnO@C_ZIF-8_ employed as an anode for the Ni-Zn secondary battery were investigated. Benefiting from the unique core-shell heterostructure consisting of the ZIF-8 inherently derived carbon shell and porous ZnO core with abundant active sites, the ZnO@C_ZIF-8_ nanocomposites present a stable base structure and improved cycling stability.

## Experiment

### Synthesis of ZIF-8

Zn (NO_3_)_2_·6H_2_O (1.1158 g) was dissolved in 30 ml methanol under ultrasonic treatment. 2-Methylimidazole (1.2337 g) was dissolved in 30 ml methanol. Then, the aforementioned solutions were mixed. The mixed solution was continuously stirred for 20 h. After that, the white precipitate was washed with methanol 3 times and vacuum dried.

### Synthesis of ZnO@ZIF-8

Zn (CH_3_COO)_2_ (6.5 g) was first hemolyzed in 300 ml diethylene glycol under ultrasonication for half of an hour to obtain a clear solution and then transferred into a flask. This mixture was heated at 150°C in an oil slot with continuous stirring for 0.5 h. During this step, the solution gradually changed from colorless to a milky white color. After the solution cooled to indoor temperature, the ZnO nanospheres were obtained and dried at 60°C for 10 h. The as-prepared ZnO nanospheres were dispersed in 30 ml methanol with 1.2337 g 2-methylimidazole and stirred for 0.5 h. The aforementioned mixture was poured into a reaction still and held at 70°C for 20 h. Then, ZnO@ZIF-8 was obtained by centrifugation at 8,000 rpm for 5 min, washed with methanol, and dried at 60°C.

### Synthesis of ZnO@C_ZIF-8_

The ZnO@ZIF-8 powders were annealed in an Ar atmosphere at 600°C for 3 h at a heating rate of 3°C min^−1^. After cooling to indoor temperature, ZnO@C_ZIF-8_ was obtained. For comparison, ZnO was prepared by the same process using a single ZIF-8 as a precursor, marked as ZnO (ZIF-8).

### Material Characterization

The crystalline structural characterization of the samples was investigated by X-ray diffraction (XRD, D8). Transmission electron microscopy (TEM, FEI Talos-F200S) and scanning electron microscopy (SEM, Zeiss Sigma 300) were used to observe the morphology and microstructure of the samples. Raman spectra were performed using an HR800 spectrophotometer with 633 nm laser excitation. The carbon content in the product was confirmed with thermogravimetric analysis (TGA) under an air atmosphere from 20 to 700°C. The specific area and porous property were measured via N_2_ adsorption/desorption isotherms (Quantachrome Autosorb-IQ3). The surface element component of the sample was determined via X-ray photoelectron spectroscopy (XPS, Thermo Scientific K-Alpha).

### Electrochemical Measurements

The ZnO@C_ZIF-8_ (active material, 80%), polyvinylidene fluoride (PVDF, 10%), and conductive carbon (10%) in N-methyl-2-pyrrolidone (NMP) solvent were mixed to obtain a mixed slurry. The as-prepared mixture was pasted on tinfoil and dried at 70°C in vacuum. The ZnO@C_ZIF-8_ anode was punched into a wafer (diameter of 10 mm). The loading mass of the electrode was 0.8∼1.0 mg. The electrochemical performances of ZnO@C_ZIF-8_ were determined by assembling CR2032 coin cells using agglomerative Ni(OH)_2_ as the cathode and a mixed solution (4 M KOH, 2 M K_2_CO_3_, and 2 M KF) as the electrolyte. A galvanostatic charge and discharge test was performed on the LAND-CT2001 batter-testing system. The cell was charged to 1.9 V and discharged to 1.5 V for a certain time. Cycle voltammogram (CV 1 mV s^−1^, voltage ambit between −1.9∼−1.0 V), electrochemical impedance spectroscopy (EIS, 10 kHz to 0.1 Hz), and Tafel plots were obtained by using an electrochemical workstation (CHI660D).

## Results and Discussion

**FIGURE 1:**
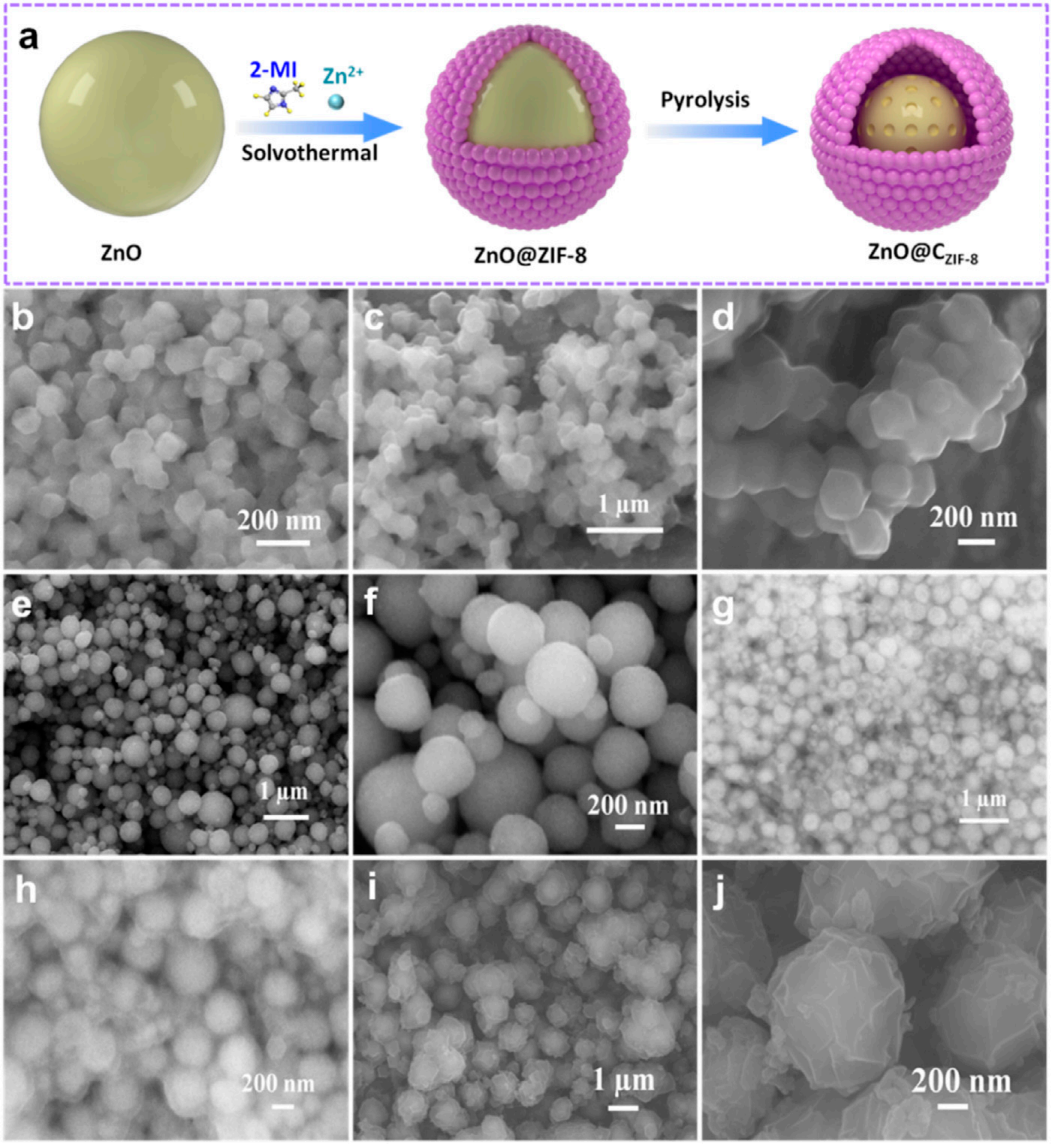
**(A)** The preparation process of core-shell ZnO@C_ZIF-8_. SEM images of **(B)** ZIF-8, **(C,D)** ZnO (ZIF-8), **(E,F)** ZnO nanosphere, **(G,H)** ZnO@ZIF-8, and **(I,J)** ZnO@C_ZIF-8_.

The core-shell structural ZnO@C_ZIF-8_ nanospheres were prepared as shown schematically in Figure 1A. First, a ZnO nanosphere precursor with a diameter range between 300 and 500 nm is synthesized by heating in an oil bath (Figures 1E,F). Second, a shell layer of ZIF-8 is intrinsically grown and coated on the surface of the nanosphere precursor by the solvothermal method. It can be observed that the ZIF-8 layer forms a coating shell on the nanosphere surface, and the obtained ZnO@ZIF-8 is uniform with a diameter size of about 600 nm (Figures 1G,H). Moreover, ZIF-8 and ZnO (pyrolysis treatment of ZIF-8) were prepared, as presented in Figures 1B–D. The ZIF-8 nanoparticles presented a rhombic dodecahedron morphology with a size of about 100 nm, and the framework structure can be maintained after the pyrolysis process. Finally, the well-designed carbon shell derived from the ZIF-8 layer can be generated and coated on the surface of the ZnO core. The inherently derived porous ZIF-8 carbon shell plays a vital role in the construction of ZnO@C_ZIF-8_. Figures 1I,J show the morphology of ZnO@C_ZIF-8_. After pyrolysis treatment, the spherical structure was preserved, and the surface became rougher, which is ascribed to the decomposition of the organic-functional groups in ZIF-8 (Li et al., 2020). The average size of ZnO@C_ZIF-8_ is about 600 nm.

**FIGURE 2:**
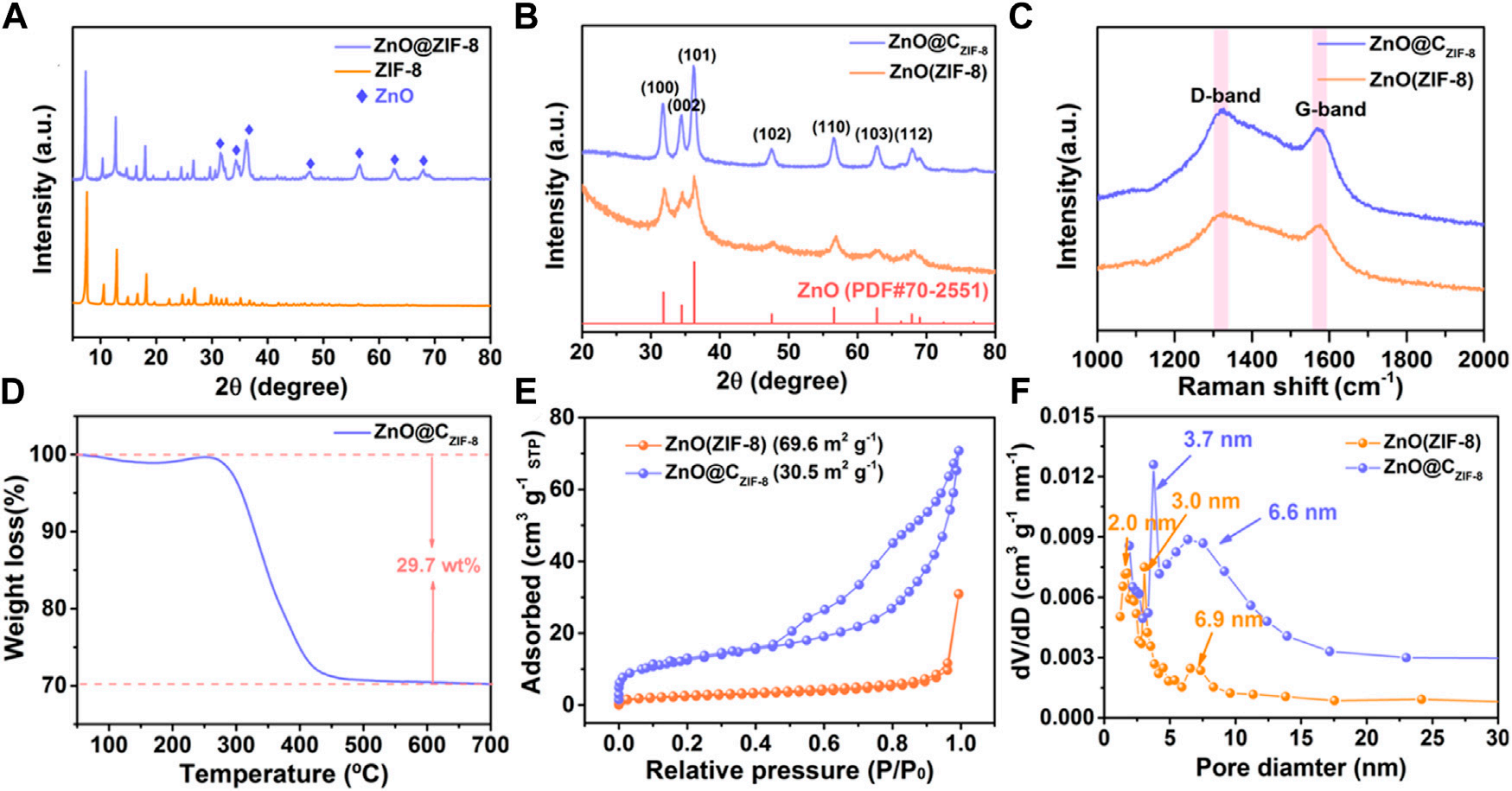
XRD patterns of **(A)** ZIF-8 and ZnO@ZIF-8, **(B)** ZnO (ZIF-8), and ZnO@C_ZIF-8_. **(C)** The Raman spectra of ZnO (ZIF-8) and ZnO@C_ZIF-8_. **(D)** The TGA curve of ZnO@C_ZIF-8_. **(E)** N_2_ adsorption–desorption isotherms and **(F)** pore size distribution curves of ZnO (ZIF-8) and ZnO@C_ZIF-8_.

In Figure 2A, the XRD pattern of ZIF-8 is consistent with the ZIF-8 crystal reported in the literature (Zhang et al., 2017) and the diffraction peaks are sharp, which indicate the high purity and great crystallinity of the material. Furthermore, the characteristic peaks of ZnO can be detected in the curve of ZnO@ZIF-8. This result confirms that the ZIF-8 layer can inherently form on the external surface of the ZnO nanosphere. All diffraction peaks of ZnO (ZIF-8) and ZnO@C_ZIF-8_ can be well matched to hexagonal ZnO (PDF^#^70-2551). The peaks at 31.8°, 34.3°, 36.6°, 47.7°, 56.5°, 62.7°, and 68.1° for ZnO (ZIF-8) and ZnO@C_ZIF-8_ were associated with the (100), (002), (101), (102), (110), (103), and (112) planes of ZnO, respectively. The Zn species in ZIF-8 can be oxidized to the metal oxide (ZnO) during pyrolysis. This is ascribed to the oxygen released from the decomposition of organic-functional groups in ZIF-8. In addition, the peaks of ZnO@C_ZIF-8_ are sharper than those of ZnO (ZIF-8), exhibiting the high crystallinity of ZnO@C_ZIF-8_.

To further inquire about the constituents and pore diameter size of the samples, Raman, TGA, and BET tests were measured. The Raman spectra for ZnO (ZIF-8) and ZnO@C_ZIF-8_ both present two distinct peaks at 1,322 cm^−1^ (D-band) and 1,575 cm^−1^ (G-band). These peaks are ascribed to disordered carbon and graphitic carbon, respectively, confirming the presence of a carbon shell (Li et al., 2017b). Figure 2A shows the TGA curves of ZnO@C_ZIF-8_ in an air atmosphere. For ZnO@C_ZIF-8_, a major weight loss appeared at 300°C due to the pyrolysis of the coated carbon shell. The carbon content in ZnO@C_ZIF-8_ is estimated to be 29.7%. Figures 2E,F present the BET curves and the pore diameter size of ZnO (ZIF-8) and ZnO@C_ZIF-8_, respectively. The specific surface areas for ZnO (ZIF-8) and ZnO@C_ZIF-8_ (Figure 2E) are estimated to be 30.5 and 69.6 m^2^g^−1^, respectively. As shown in Figure 2F, the pore diameter distributions are mostly centered at 2∼10 nm for ZnO (ZIF-8) and ZnO@C_ZIF-8_. The result indicates that the samples mainly comprise a mesoporous structure (2–50 nm). The formation of mesoporous structure for ZnO@C_ZIF-8_ is ascribed to the release of gas-phase compounds in the ZIF-8 during the carbonization (Li et al., 2020). The structural characteristics of mesoporous are helpful for the transportation of Li^+^ ions and the improvement of the active site.

**FIGURE 3:**
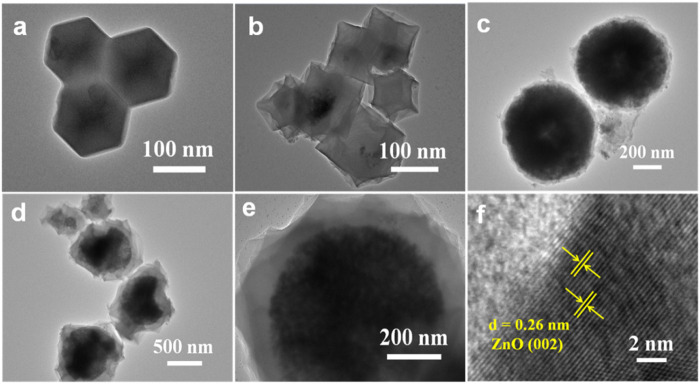
TEM images of **(A)** ZIF-8, **(B)** ZnO (ZIF-8), **(C)** ZnO@ZIF-8, **(D,E)** ZnO@C_ZIF-8_, and **(F)** HRTEM of ZnO@C_ZIF-8_.

The microstructure of the products were also investigated by TEM. The ZIF-8 particles display a uniform rhombic dodecahedron (Figure 3A). Compared with ZIF-8, the surface of ZnO (ZIF-8) is sunken and shrunken after carbonization (Figure 3B), and the particle size is slightly reduced. Agglomeration occurs between the particles for both ZIF-8 and ZnO (ZIF-8). As presented in Figure 3C, ZnO@ZIF-8 exhibits a sphere-shaped heterostructure coated with a ∼50 nm inherent growth of the ZIF-8 shell, and the particle size of ZnO@ZIF-8 is ∼700 nm. Figures 3D,E show the TEM images of ZnO@C_ZIF-8_. The microsphere structure can be maintained after carbonization. The carbon-shell–derived ZIF-8 layer is coated on the external face of the ZnO core. Furthermore, the pyrolysis of the coated ZIF-8 layer can cause volume contraction of ZnO@C_ZIF-8_. Thus, the external shell of ZnO@C_ZIF-8_ becomes rough, and the particle size decreases. Agglomeration can be controlled, owing to the preservation of the carbon shell. The HRTEM image of ZnO@C_ZIF-8_ (Figure 3F) presents lattice fringes with an interplanar spacing of 0.26 nm, matching the (002) plane of ZnO.

**FIGURE 4:**
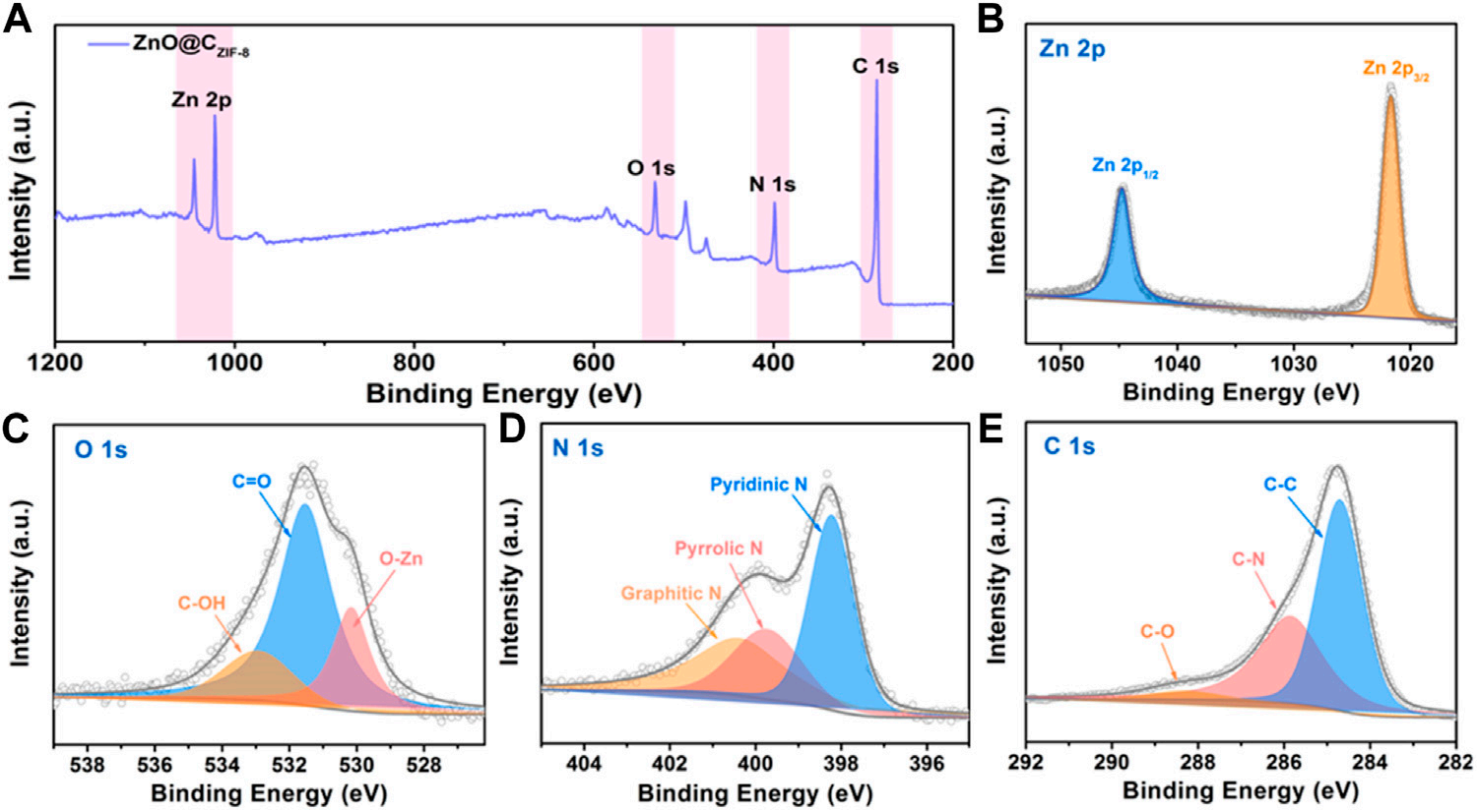
**(A)** The Survey XPS spectrum of ZnO@C_ZIF-8_ microsphere. **(B–E)** High-resolution XPS spectra of Zn 2p, C 1s, O 1s, and N 1s.

The surface element compositions and valences of the as-prepared ZnO@C_ZIF-8_ were analyzed using XPS. The full spectrum in Figure 4A shows the presence of Zn, N, O, and C elements in ZnO@C_ZIF-8_. The Zn 2p spectrum of ZnO@C_ZIF-8_ contains two characteristic peaks at 1,043.8 and 1,022.1 eV, matching Zn 2p1/2 and Zn 2p2/3, respectively. This result reveals the existence of a Zn (II) oxidation state in ZnO@C_ZIF-8_. For the O 1s spectrum of ZnO@C_ZIF-8_ (Figure 4D), the peak is fitted for three peaks at 533.1, 531.7, and 530.1 eV, respectively. The characteristic peak at 530.1 eV is matched to the lattice oxygen of ZnO, and the other two peaks at 533.1 and 531.7 eV are derived from the C-OH and C=O in the carbon shell, respectively (Zeng et al., 2019b). The N 1s spectrum of the ZnO@C_ZIF-8_ is presented in Figure 4D. The broadband is fitted into three peaks, which are ascribed to graphitic-N (400.5 eV), pyrrolic-N (399.7 eV), and pyridinic-N (298.1 eV), respectively, derived from the splitting decomposition of the organic-functional group in the ZIF-8 layer during carbonization. As is well known, N-doped graphitized carbon can be used as additional active sites to improve zinc storage (Xu et al., 2022). The C 1s spectrum of ZnO@C_ZIF-8_ is also given (Figure 4C). The C 1s spectrum can be fitted into three spectral peaks, assigned to C-O (288.1 eV), C-N (285.9 eV), and C-C (284.6 eV). The formation of C-N bands reveals that N-atoms are anchored on the carbon shell. Moreover, the existence of N-doped carbon can also enhance the electrical conductivity of the base material (Xu et al., 2022).

**FIGURE 5:**
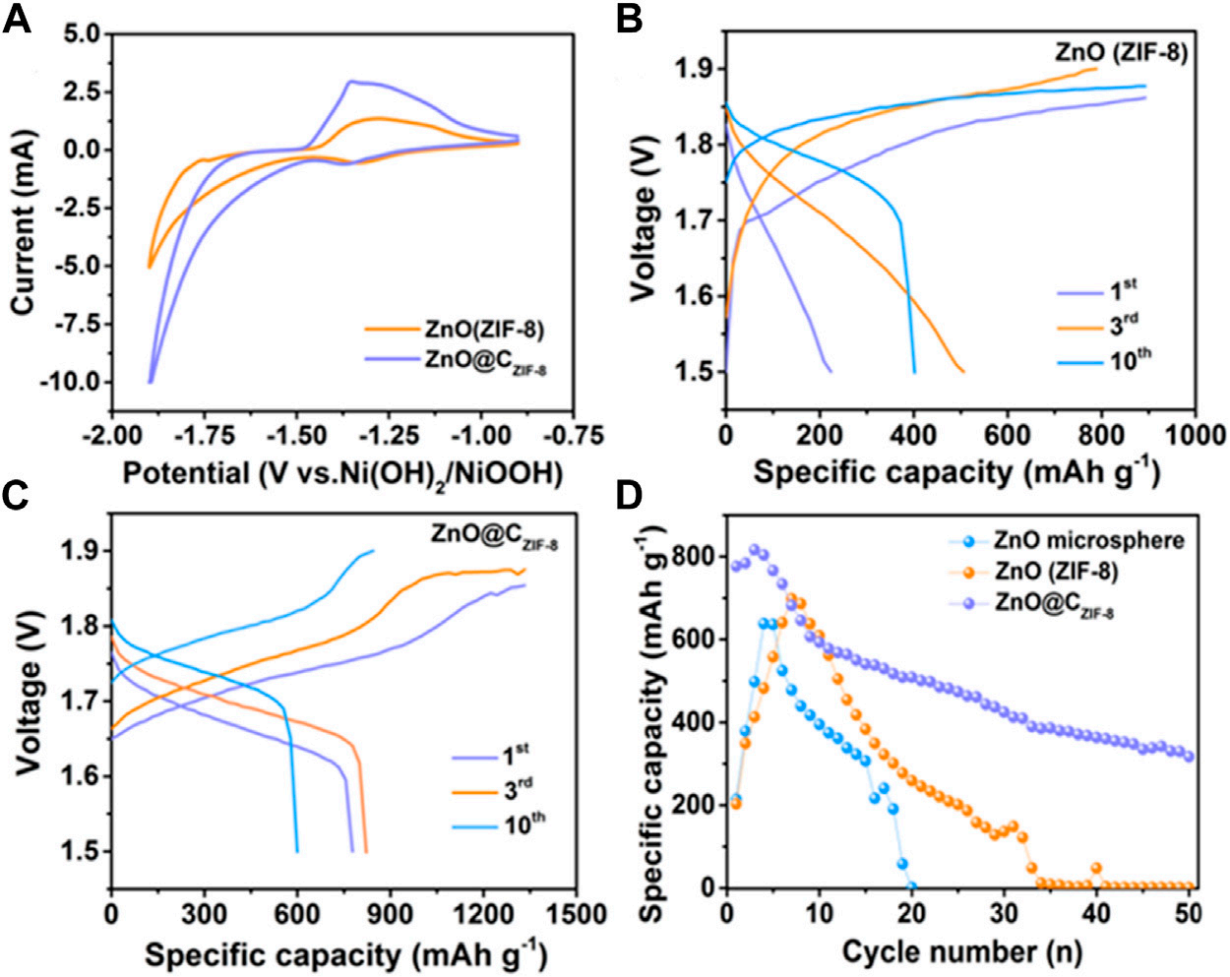
**(A)** Cyclic voltammogram curves of ZnO (ZIF-8) and ZnO@C_ZIF-8_. Galvanostatic charge and discharge curves of **(B)** ZnO (ZIF-8) and **(C)** ZnO@C_ZIF-8_ at 1st, 3rd, and 10th cycles. **(D)** Cycling performance of ZnO microsphere, ZnO (ZIF-8), and ZnO@C_ZIF-8_ at 1 A g^−1^.

The electrochemical performances of the as-prepared samples were tested by constructing a button cell using commercial sintered Ni (OH)_2_ as the cathode, as shown in Figure 5. To confirm the related electrochemical behaviors during the discharge–charge processes, a cycling voltammogram (CV) was tested with a voltage window amid −1.9 and −1.0 V at a scan rate of 1 mV s^−1^. It can be observed that all electrodes show similar CV curves, which include the reduction peaks for ZnO@C_ZIF-8_ (−1.37 V) and ZnO (ZIF-8) (−1.34 V) and the oxidation peaks for ZnO@C_ZIF-8_ (−1.35 V) and ZnO (ZIF-8) (−1.29 V). The potential intervals between the oxidation peak and the reduction peak of ZnO (ZIF-8) and ZnO@C_ZIF-8_ are 0.046 and 0.03 V, respectively. The lower potential interval implies that the ZnO@C_ZIF-8_ anode presents better reversibility (Yan et al., 2018). The electrochemical reactions can be presented as follows:

$$\text{Charge process: }ZnO+2OH^{-}+H_{2}O\to Zn{\left(OH\right)}_{4}^{2-},$$ (1)

$$Zn{\left(OH\right)}_{4}^{2-}+2e^{-}\to Zn+4OH^{-},$$ (2)

$${\text{Discharge process: Zn}+4\text{OH}}^{-}\to Zn{\left(OH\right)}_{4}^{2-}+2e^{-},$$ (3)

$$Zn{\left(OH\right)}_{4}^{2-}\to Zno+2OH^{-}+H_{2}O.$$ (4)

Figures 5B,C show the discharge and charge curves of ZnO (ZIF-8) and ZnO@C_ZIF-8_ at different cycles (1st, 3rd, and 10th cycles). The discharge specific capacities of ZnO@C_ZIF-8_ are 777, 820, and 601 mAh g^−1^ at the 1st, 3rd, and 10th cycles, respectively, which are larger than those of ZnO (ZIF-8) (223, 507, and 401 mAh g^−1^ at the first, third, and 10th cycles). For comparison, the cycling performances of ZnO@C_ZIF-8_, ZnO (ZIF-8), and ZnO (nanosphere) are presented in Figure 5D. The specific capacity of ZnO (nanosphere) declined seriously and depleted after 20 cycles. The ZnO (ZIF-8) anode suffers the same experience. In contrast, the cycling performance of ZnO@C_ZIF-8_ remained steady, and the discharge capacity reached 316 mAh g^−1^ after 50 cycles. This benefit stemmed from the synergistic effect of the carbon shell derived from the inherent ZIF-8 layer and ZnO nanoparticle core.

**FIGURE 6:**
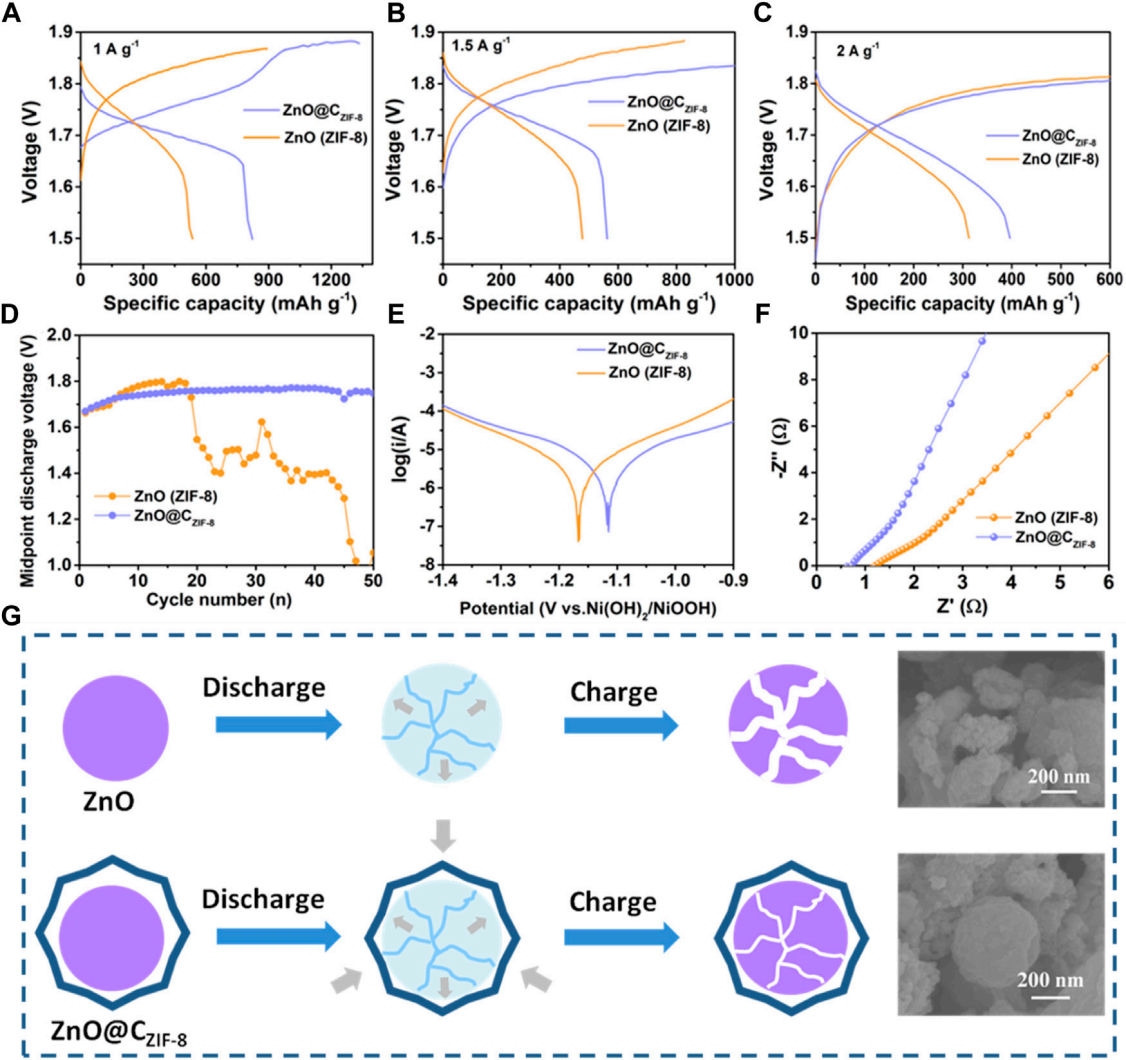
Rate performance of ZnO (ZIF-8) and ZnO@C_ZIF-8_ electrodes at different current densities: **(A)** 1 A g^−1^, **(B)** 1.5 A g^−1^, and **(C)** 2 A g^−1^. **(D)** Midpoint discharge voltage curves of the Ni-Zn batteries with different anodes of ZnO (ZIF-8) and ZnO@C_ZIF-8_. **(E)** The Tafel plot of ZnO (ZIF-8) and ZnO@C_ZIF-8_ electrodes. **(F)** Nyquist plots of ZnO (ZIF-8) and ZnO@C_ZIF-8_ electrodes. **(G)** Morphological changes after the charge/discharge processes.

Figures 6A–C present the rate stabilities of ZnO (ZIF-8) and ZnO@C_ZIF-8_ at various current densities. The discharge specific capacities of ZnO@C_ZIF-8_ at 1, 1.5, and 2 A g^−1^ are 821, 562, and 396 mAh g^−1^, respectively, which are larger than those of ZnO (ZIF-8) (536, 477, and 312 mAh g^−1^ at 1, 1.5, and 2 A g^−1^). Figure 6D displays the midpoint discharge voltage charts of ZnO (ZIF-8) and ZnO@C_ZIF-8_, which is also a significant argument for rechargeable batteries. The better the stability and higher the midpoint discharge voltage, the higher the specific energy and the greater the electrochemical property. ZnO@C_ZIF-8_ exhibits a stable and high midpoint discharge voltage during cycling. However, the midpoint discharge voltage of ZnO (ZIF-8) markedly decreases after 18 cycles. The Tafel plot curves (Figure 6E) of ZnO (ZIF-8) and ZnO@C_ZIF-8_ are exhibited to investigate the anticorrosion performance of the electrode in alkaline solution, assessed using corrosion potential (E_corr_) (Li et al., 2017a). We observed that the value of E_corr_ for ZnO@C_ZIF-8_ (−1.115) was more positive than that of ZnO (ZIF-8) (−1.167). This indicates that the ZnO@C_ZIF-8_ electrode exhibits better corrosion resistance. The mainspring was that the coated carbon shell can control the corrosion of ZnO. Nyquist plots of the ZnO (ZIF-8) and ZnO@C_ZIF-8_ electrodes are exhibited in Figure 6F. All plots are semi-circular in the high-frequency region and show an oblique stroke in the low-frequency region. These are related to charge transfer and ion diffusion in the electrode. Obviously, the semi-circular diameter of ZnO@C_ZIF-8_ is smaller than that of ZnO (ZIF-8), implying that the coated carbon shell enhances the electronic conductivity of the base material. The morphological changes of ZnO and ZnO@C_ZIF-8_ after the cycles are presented in Figure 6G. The ZnO (ZIF-8) suffers an inevitable volume increase during cycling, causing fracture of the material. By constructing the inherently derived core-shell structure, the ZIF-8–derived carbon shell restricts the volume expansion of the ZnO core during the cycling process. This indicates that the inherently derived carbon shell clings to the surface of ZnO and effectively ensnares the volume expansion of the active material, thereby increasing the cycling performance. The superior electrochemical performances of ZnO@C_ZIF-8_ can be ascribed to its unique hierarchical structure. First, the microsize of ZnO@C_ZIF-8_ guarantees more efficient infiltration between the electrolyte and the electrode. Second, the existence of a carbon shell derived from the inherent ZIF-8 layer can not only weaken the dissolution of ZnO and be the detriment of zinc dendrites but can also increase the electronic conductivity of the electrode.

## Conclusion

In summary, a unique core-shell ZnO@C_ZIF-8_ nanocomposite was successfully synthesized using a ZnO nanosphere as the core and an inherent ZIF-8 layer as the coated carbon source by using a simple hydrothermal method and subsequent pyrolysis process. The inherent ZIF-8–derived carbon shell with N-doping can improve the electronic conductivity and offer abundant active sites. Meanwhile, this hierarchical structure provides an extreme self-adaptive framework that can efficiently control the volume expansion of the electrode. Benefiting from the unique hierarchical structure, the ZnO@C_ZIF-8_ nanocomposite exhibits superior electrochemical properties when used as anode material in the Ni-Zn secondary battery. In particular, the ZnO@C_ZIF-8_ electrode presents a discharge-specific capacity of 820 mAh g^−1^, which is larger than that of the ZnO (ZIF-8) (507 mAh g^−1^) and ZnO (nanosphere) precursor (410 mAh g^−1^). In addition, the ZnO@C_ZIF-8_ presents remarkable cycling stability and outstanding rate stability. The advanced electrochemical performances of the ZnO@C_ZIF-8_ electrode can be attributed to the conductivity improvement, structure stability, anticorrosion property, and reaction reversibility of the inherent combination between the carbon shell and ZnO core. Therefore, this study offers a guide to constructing hierarchical inherent carbon-coated ZnO with outstanding electrochemical performances.

## Data Availability

The original contributions presented in the study are included in the article/Supplementary Material; further inquiries can be directed to the corresponding author.
